# Functional consequences of *DECTIN-1 *early stop codon polymorphism Y238X in rheumatoid arthritis

**DOI:** 10.1186/ar2933

**Published:** 2010-02-16

**Authors:** Theo S Plantinga, Jaap Fransen, Nozomi Takahashi, Rinke Stienstra, Piet L van Riel, Wim B van den Berg, Mihai G Netea, Leo AB Joosten

**Affiliations:** 1 Department of Medicine, Radboud University Nijmegen Medical Centre, P.O. Box 9101, 6500 HB Nijmegen, The Netherlands; 2 Nijmegen Institute for Infection, Inflammation and Immunity (N4i), Radboud University Nijmegen Medical Centre, P.O. Box 9101, 6500 HB Nijmegen, The Netherlands; 3 Department of Rheumatology, Radboud University Nijmegen Medical Centre, P.O. Box 9101, 6500 HB Nijmegen, The Netherlands; 4 Rheumatology Research and Advanced Therapeutics, Radboud University Nijmegen Medical Centre, P.O. Box 9101, 6500 HB Nijmegen, The Netherlands; 5 Molecular Signalling and Cell Death Unit, Department for Molecular Biomedical Research, Ghent University, VIB Research Building FSVM, Technologiepark 927, 9052 Ghent, Belgium; 6 Department of Biomedical Molecular Biology, Ghent University, Technologiepark 927, 9052 Ghent, Belgium

## Abstract

**Introduction:**

Dectin-1, a pattern recognition receptor expressed by the innate immune system, is known to be a major receptor inducing Th17-type adaptive immune responses that have been demonstrated to mediate autoimmunity. In this study, dectin-1 mRNA and protein expression, as well as the recently characterized *DECTIN-1 *Y238X early stop codon polymorphism, were studied in relation to rheumatoid arthritis (RA) susceptibility and severity.

**Methods:**

Dectin-1 mRNA expression was measured in synovial tissue specimens of RA, osteoarthritis (OA), and nonrheumatic patients. Dectin-1 protein expression and localization were assessed in RA synovial tissue specimens. Macrophages from individuals with different *DECTIN-1 *genotypes were examined for differences in cytokine responses on dectin-1 stimulation. Furthermore, clinical parameters of inflammation and bone destruction of 262 RA patients were correlated with the presence of the *DECTIN-1 *Y238X polymorphism.

**Results:**

Evaluation of dectin-1 mRNA expression in synovial tissue biopsies revealed an increased expression in RA specimens, compared with biopsies from OA and nonrheumatic patients. Accordingly, dectin-1 protein expression in RA synovial tissue biopsies was moderate to high, especially on macrophage-like cells. Cytokine production capacity of macrophages bearing the *DECTIN-1 *Y238X polymorphism was demonstrated to be impaired on dectin-1 stimulation. However, the presence of the *DECTIN-1 *Y238X polymorphism was not associated with RA susceptibility or disease severity.

**Conclusions:**

Although expression of dectin-1 was high in synovial tissue of RA patients, and reduced cytokine production was observed in macrophages of individuals bearing the *DECTIN-1 *Y238X polymorphism, loss of one functional allele of *DECTIN-1 *is not associated with either susceptibility to or severity of RA.

## Introduction

Rheumatoid arthritis (RA) is a chronic inflammatory disorder that results in severe cartilage damage and bone destruction in synovial joints. Despite unclear disease etiology, it is commonly appreciated that both genetic and environmental factors are underlying risk factors in the pathogenesis of RA. In recent years, an important role for innate immune receptors in RA has emerged, especially focused on members of the Toll-like receptor (TLR) family [1,2]. These innate responses were recently suggested to modulate and induce the autoimmune-related Th17 responses [3,4].

A different class of innate immune receptors involved in microbial recognition and subsequent immune signalling are C-type lectins, of which dectin-1 is one of the most well characterized members. After its discovery as a receptor for fungal-derived 1,3-β-glucans [5], its intracellular signalling has been demonstrated to be mediated by Raf-1 and Syk-CARD9 dependent pathways to induce production of pro-inflammatory cytokines and reactive oxygen species [6-10]. Other studies have uncovered that dectin-1 converges with TLR signalling [11,12] for the induction of cytokine responses and is able to promote Th17 and cytotoxic T-cell responses through activation of dendritic cells [13,14]. It has been well established that fungal particles, either intact yeast or fungal cell wall components that can be recognized by dectin-1, such as zymosan, can act as adjuvants in several experimental models of RA [15-19]. In addition, a study by Yoshitomi and colleagues [20] revealed that β-glucan induced autoimmune arthritis in genetically susceptible SKG mice could be prevented by blocking the dectin-1 receptor.

These studies imply that dectin-1 plays a pivotal role in the innate immune system and is able to modulate adaptive immune responses, of which, especially Th17 responses are implicated in immunopathology. Furthermore, dectin-1 is involved in the induction of arthritis in mouse models through induction of intracellular signalling on recognition of fungal components. As a consequence, dectin-1 mediated inflammatory responses could contribute to the aetiology or disease severity of RA.

Recently we characterized an early stop codon polymorphism Y238X (c.714T>G, rs16910526) in *DECTIN-1 *[21], which was demonstrated to result in a complete loss of function of the protein. Cytokine production capacity of peripheral blood mononuclear cells (PBMCs) from individuals homozygous for the *DECTIN-1 *Y238X polymorphism on β-glucan or *Candida albicans *exposure are impaired, including TNF-α, interleukin (IL-)1β, IL-6, and IL-17 responses. In the same stimulation assays, individuals heterozygous for the *DECTIN-1 *Y238X polymorphism exhibited intermediate cytokine responses compared with wild-type individuals [22].

Considering both the involvement of dectin-1 in pro-inflammatory responses and the significant consequences of the Y238X polymorphism for dectin-1 function, it is compelling to assess whether dectin-1 and the *DECTIN-1 *Y238X polymorphism play a role in the pathogenesis or disease severity of RA. In the present study, mRNA expression of dectin-1 was assessed in synovial tissue biopsies obtained from RA patients and compared with synovial tissue specimens from osteoarthritis (OA) patients and from patients with other underlying joint pathology not related to RA. In addition, dectin-1 protein expression was assessed in tissue sections of synovial lesions obtained from RA patients. The functional consequences of the presence of the *DECTIN-1 *Y238X polymorphism on cytokine production capacity of macrophages were studied by stimulating the cells with β-glucans. Furthermore, the presence of the *DECTIN-1 *Y238X polymorphism was correlated with disease susceptibility in a cohort of 262 RA patients, and within this cohort, clinical parameters of joint inflammation and bone destruction were compared after stratifying for the *DECTIN-1 *genotype.

## Materials and methods

### Patients

For assessing the effect of the *DECTIN-1 *Y238X polymorphism on the disease course, patient data were used from the early RA inception cohort at our clinic, described in more detail elsewhere [23]. Patients were included in this cohort if they fulfilled the ACR (American College of Rheumatology) classification criteria for RA, were at least 18 years old, had a disease duration not exceeding 1 year, and did not use DMARDs or biologic response modifiers. Age, gender, and IgM rheumatoid factor were determined at baseline. At baseline and every 3 months thereafter, patients were assessed by specialized research nurses who assigned joint inflammation scores and drew a blood sample for determination of the erythrocyte sedimentation rate. The patients indicated their global disease activity on a Visual Analogue Scale. These data were used to calculate the disease activity score (DAS28) according to the original formula [24]. Radiographs of the hand and feet were made at baseline, year 1, 2, and 3, and every third year thereafter. Radiographs of hands and feet were read in chronologic order by one of four raters, according to the Ratingen score by using reference pictures [25]. The Ratingen score (range, 0-190) is a modification of the Larsen score and evaluates joint surface destruction, graded from 0 to 5, in 38 hand and feet joints, separately. The interrater reliability was ICC = 0.85, tested previously with the four raters in 10 patients over 9 years. Clinical data were entered in a computerized database.

From 2006 to 2008, additional blood was collected in a convenience sample of the cohort, used for genotyping for the *DECTIN-1 *Y238X polymorphism. For the current study, data from cohort patients were included if a blood sample was available with a joint damage assessment at year 3.

Consequently, 262 patients were included. The study was approved by our institutional review board, and informed consent of the patients was obtained before enrollment. The study was performed according to the principles of the Declaration of Helsinki.

### RNA isolation from synovial tissue

Synovial tissue samples of RA, OA, and nonrheumatic patients were dissected during surgery or by fine needle arthroscopy under camera supervision. The tissue samples were stored at a tissue bank under liquid nitrogen until further processing. Total RNA was isolated and purified on an affinity resin (RNeasy Kit for fibrous tissues, Qiagen, Valencia, CA, USA) according to the manufacturer's instructions. Quantity and purity were assessed by using Agilent bioanalyzer (Agilent Technologies, Santa Clara, CA, USA), and integrity, by using nanodrop (Thermo Scientific, Waltham, MA, USA) according to the manufacturer's instructions. Total RNA was stored at -80°C until further processing.

### Oligonucleotide array

To measure dectin-1 mRNA expression, 100 ng of total RNA was used as starting material for cDNA preparation. A two cycle amplification protocol was followed. Generation of biotinylated cRNA and subsequent hybridization to U133Plus 2.0 oligonucleotide arrays (Affymetrix, Santa Clara, CA, USA), washing, and staining were performed according to Affymetrix Expression Analysis Technical Manual for two cycle amplification [26]. The arrays were then scanned by using a laser scanner GeneChip^® ^Scanner (Affymetrix) and analyzed by using Affymetrix GeneChip Operating Software (GCOS version 1.4) according to the manufacturer's instructions. Array normalization and model-based calculation of expression values were performed by using DNA-Chip Analyzer (dChip) version 1.3 [27]. The Invariant Set Normalization method and the model based method were used for computing expression values [28]. These values were expressed as mean and standard error (SE).

### Quantitative RealTime PCR

RNA samples were reverse transcribed by using oligo-dT primers and MMLV reverse transcriptase. Primers were designed with Primer Express (Applied Biosystems, Foster City, CA, USA). Q-PCR was performed by using the ABI Prism 7000 sequence detection system (Applied Biosystems) for an amount of 10 ng cDNA with SYBR Green Master mix. Quantification of the PCR signals was performed by comparing the cycle threshold value (C_t_) of the gene of interest of each sample with the C_t _values of the reference gene GAPDH (ΔCt), and expressed as 2^-ΔCt ^multiplied by arbitrary factor. Fold change was calculated as the mean ratio between the relative transcript levels. The sequences of primer sets used were as follows: 5'-TTCCCCATGGTGTCTGAGC-3' (GAPDH forward), 5'-ATCTTCTTTTGCGTCGCCAG-3' (GAPDH reverse), 5'-TGACTCCTACCAAAGCTGTCAAAAC-3' (dectin-1 forward), and 5'-TTCTCATATATAATCCAATTAGGAGGACAAG-3' (dectin-1 reverse).

### Immunohistochemical staining in synovial tissue

In specimens obtained from knee surgery, dectin-1 protein expression was evaluated by immunohistochemical staining in paraffin-embedded inflamed synovial tissue sections of RA patients. The applied primary antibody was a monoclonal mouse-anti-human dectin-1 antibody (MAB 1859, purchased from R&D Systems, Minneapolis, MN, USA), used in a concentration of 5 μg/ml. After overnight incubation with the primary antibody, the tissue sections were incubated for 1 h with a secondary antibody after washing with PBS. Subsequently, the staining was visualised by applying ABC complex and DAB solution. Sections were counterstained with haematoxylin. Staining with a mouse IgG2b isotype control antibody served as a negative control.

### *In vitro *macrophage stimulation assays

PBMCs were obtained from healthy donors, either wild-type or heterozygous for the Y238X polymorphism. Cells homozygous for the *DECTIN-1 *Y238X polymorphism were obtained from three members of a family previously analyzed for mucocutaneous *Candida *infections [22]. PBMCs were isolated from peripheral blood as described previously [11]. The PBMC fraction was plated in flat-bottom 96-well plates. After 4 h of culture at 37°C, cells were washed 3 times with culture medium, and the nonadherent cells were removed. The adherent monocytes were cultured for 6 days in culture medium with 10% heat-inactivated pooled human serum, until the monocytes exhibited macrophage-like morphology and expressed characteristic surface markers analyzed with flow cytometry. On day 6 of culture, after washing 3 times with fresh medium, macrophages were stimulated for 24 h with β-glucan (10 μg/ml), Pam3Cys (10 μg/ml), or with a combination of the two stimuli. Cytokine production was measured with ELISA (purchased from R&D Systems) according to the guidelines of the manufacturer. Detection levels were 10 pg/ml for TNF-α and 20 pg/ml for IL-1β.

### Genotyping for *DECTIN-1 *Y238X polymorphism

Genomic DNA was isolated from peripheral venous blood by using standard techniques and stored at 4°C. Genotyping for the presence of the Y238X polymorphism in exon 6 of the *DECTIN-1 *gene (also known as *CLEC7A*) in the patient and in healthy control groups was performed by applying the predesigned TaqMan SNP assay C_33748481_10 on the 7300 ABI Real-Time PCR system (both from Applied Biosystems). We declare that all the subjects included in this study were prospectively asked to provide consent in regard to the use of clinical data as well as DNA samples for future investigations. All patients gave informed consent, as required by our local ethics committee and in accordance with the Declaration of Helsinki.

### Statistics

Statistical analysis for the oligonucleotide array-based gene expression was performed by using dChip. The *t *statistic was computed as (mean1 - mean2)/); its value is computed based on the *t *distribution, and the degree of freedom is set according to Welch modified two sample *t *test [28]. Statistical analysis of the gene expression data obtained by quantitative PCR and of the cytokine measurements obtained with ELISA was performed by applying the Mann Whitney *U *test.

Concerning the correlation of the *DECTIN-1 *genotype with clinical RA parameters, the following statistical tests were applied. Between-group differences between *DECTIN-1 *wild-type and heterozygous patients were analyzed by using a χ^2 ^test, a *t *test or a Wilcoxon test, as appropriate. To test the effect of the *DECTIN-1 *genotype on the progression of joint damage, for every patient, the annual joint-damage progression rate was calculated by subtracting the last available joint damage score from the baseline joint damage score, and dividing the joint damage progression by follow-up time. The difference between wild-type and heterozygous patients was tested by using a linear regression model with uptake of confounders. Regression assumptions were tested by using residual plots and predicted-versus-observed plots. The analysis was repeated by using longitudinal regression analysis (mixed models), by using all available data while correcting for repeated measurements within patients.

For all statistical analyses, a *P *value < 0.05 was considered significant.

## Results

### Dectin-1 mRNA and protein expression in synovial tissue

**Figure 1:**
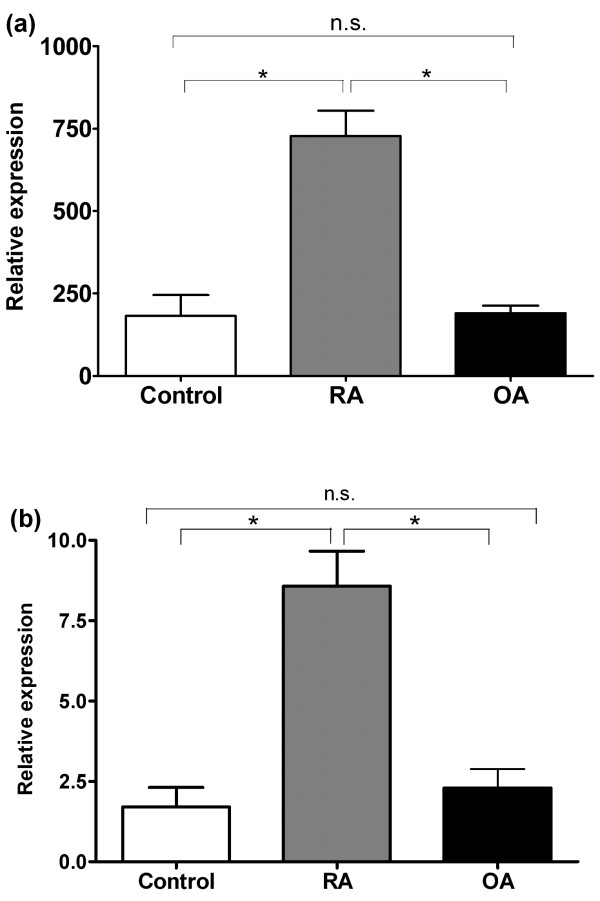
**(a) Dectin-1 mRNA expression of human synovial tissue obtained from six nonrheumatic control individuals, 20 rheumatoid arthritis patients (RAs), and 10 osteoarthritis patients (OAs)**. Dectin-1 mRNA expression was analyzed with oligonucleotide array (Affymetrix system). Values represent computed expression values. **(b) **Confirmation of microarray data by qPCR. Data are based on four control samples, seven samples obtained from RA patients, and 6 samples from OA patients. Relative expression is depicted compared with expression of the housekeeping gene GAPDH. Data are expressed as mean ± SD; *P ≤ 0.05; n.s., not significant.

**Figure 2:**
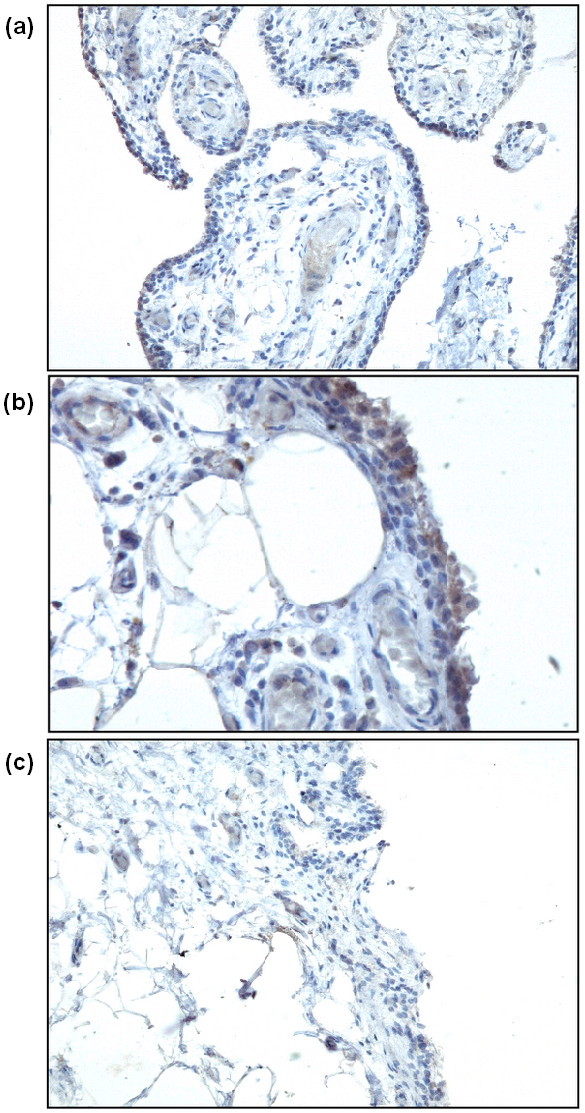
**Immunohistochemical staining for dectin-1 on paraffin-embedded synovial tissue specimens obtained from rheumatoid arthritis (RA) patients**. Pictures are representative of staining on synovial tissue biopsies from five patients. **(a, b) **anti-dectin-1 staining; **(c) **isotype control antibody. Original magnification: (a and c) 200×; and (b) 400×.

To gain insight into the distribution and amount of dectin-1 expression in human synovial tissue, dectin-1 mRNA expression was measured in synovial tissue from RA, OA, and nonrheumatic patients with an oligonucleotide array and reevaluated with quantitative PCR. Microarray analysis revealed a 4-times elevated mRNA expression in RA synovial lesions compared with OA and nonrheumatic synovial tissues (Figure 1a). These findings were confirmed with quantitative PCR (Figure 1b). Furthermore, synovial biopsies from RA patients were immunohistochemically stained for dectin-1 protein expression. Dectin-1 protein appeared to be moderately to highly expressed in RA lesions and was preferentially expressed on the membranes of macrophage-like cells that infiltrated into the synovial tissue, which were present in the synovial sublining and in close proximity to blood vessels (Figure 2).

### *In vitro *macrophage stimulation assays

**Figure 3:**
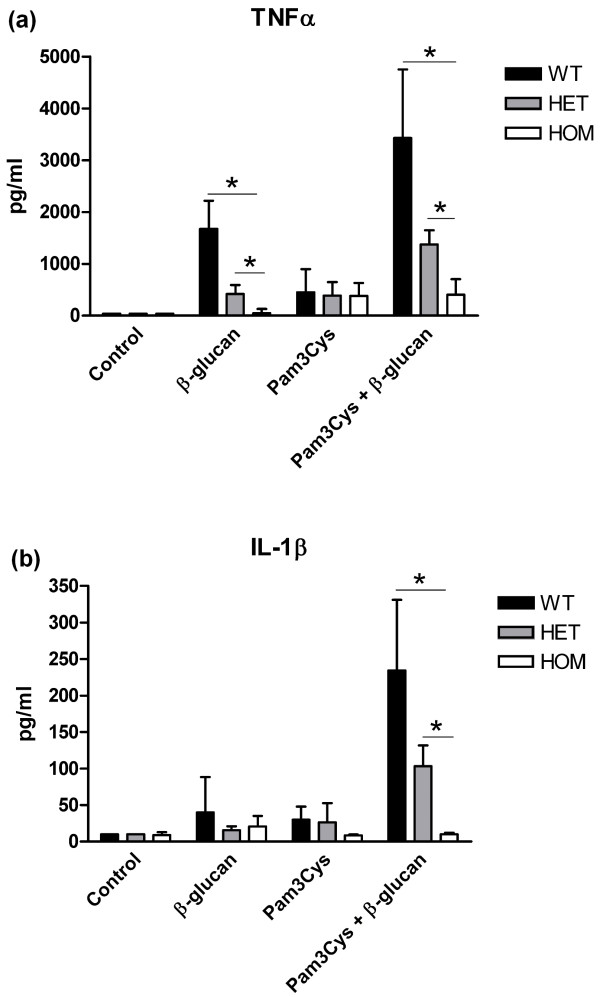
**Cytokine production capacity of TNF-α (a) and IL-1β (b) after stimulation of monocyte derived macrophages during 24 hours with β-glucan, Pam3Cys, or β-glucan/Pam3Cys**. Cells were obtained from individuals with the wild-type (WT, n = 6), heterozygous (HET, n = 4), and homozygous (HOM, n = 4) for the *DECTIN-1 *Y238X polymorphism. Cytokine concentrations were determined with enzyme-linked immunosorbent assay (ELISA). Data are expressed as mean values ± SD, *P ≤ 0.05.

Because especially macrophages are known to express dectin-1 in high amounts and are possibly involved in RA pathogenesis, we analyzed the functional consequences of the *DECTIN-1 *Y238X polymorphism for the inflammatory response of these cells with dectin-1 stimulation. Monocytes were differentiated into macrophages *in vitro *and were stimulated for 24 hours with β-glucan, the TLR2 agonist Pam3Cys, and both ligands simultaneously. After stimulation with β-glucan, cytokine measurements revealed a diminished TNF-α and IL-1β production capacity in cells from individuals homozygous for the Y238X polymorphism compared with cells from wild-type individuals. In cells from heterozygous individuals, these responses were intermediate. Moreover, the previously described synergy between dectin-1 and TLR2 induced responses [11,12] regarding TNF-α and IL-1β production was abolished in cells isolated from individuals with the polymorphism. The TLR2/dectin-1 synergism was reduced in cells isolated from heterozygous individuals and was completely absent in cells obtained from individuals homozygous for the Y238X polymorphism compared with the individuals bearing only the wild-type *DECTIN-1 *allele (Figure 3).

### Genotyping of RA patients compared with healthy controls

To assess whether the *DECTIN-1 *Y238X polymorphism is associated with an altered susceptibility to RA, a cohort of 262 RA patients and a cohort of healthy individuals (n = 284) were screened for the presence of the polymorphism. The allele frequency of the polymorphism was 7.8% in the RA cohort, compared with 7.6% in the cohort of healthy individuals (*P *= 0.87). All individuals bearing the polymorphism were heterozygous (Table 1).

**Table 1:** Genetic distribution of the *DECTIN-1 *Y238X polymorphism in a patient cohort of rheumatoid arthritis (n = 262) and in a group of healthy controls (n = 284)

	*DECTIN-1 *genotype			Allele frequency	
**Cohort**	**Wild-type**	**Heterozygous**	**Homozygous**	**Wild-type**	**Derived**

RA (n = 262)	84.4% (221)	15.6% (41)	0	92.2%	7.8%
Controls (n = 284)	84.9% (241)	15.1% (43)	0	92.4%	7.6%

RA: rheumatoid arthritis.

### Effects of *DECTIN-1 *genotype on clinical parameters of rheumatoid arthritis

The clinical data of the 262 cohort patients are shown in Table 2. At the different time points, no differences were seen in joint damage and a tendency for higher DAS28 values between the patients with a heterozygous or wild-type *DECTIN-1 *genotype. Follow-up time was similar in both genotype groups; 50% were followed up for 9 years, whereas 70% were followed up for at least 6 years. The mean annual joint damage progression rate was 3.33 per year in patients bearing the wild-type *DECTIN-1 *compared with 3.38 per year in patients heterozygous for the *DECTIN-1 *Y238X allele. The uncorrected between-group difference was nearly zero, with an estimated mean annual joint damage progression of 0.05 with *P *= 0.95 (Table 3). When corrected for joint damage at baseline, rheumatoid factor positivity, and the average DAS28 as possible confounders, the between-group difference remained insignificant (*P *= 0.57). Regression assumptions were met. The results of the longitudinal regression analysis (mixed models) were not different (not shown).

**Table 2:** Joint inflammation and bone destruction

Variable	*n*	Homozygous wild-type for *DECTIN-1*	*n*	Heterozygous for *DECTIN-1 *Y238X	*P *value
Age (years)	221	53 (14)	41	53 (13)	0.87
Female	221	147 (66%)	41	25 (61%)	0.49
Rheumatoid factor +	220	163 (74%)	41	34 (83%)	0.23
DAS28 baseline	212	5.2 (1.5)	40	5.3 (1.4)	0.55
Average DAS28 year 0-3	214	3.9 (1.1)	41	4.1 (1.2)	0.37
Average DAS28 year 4-6	203	3.5 (1.1)	35	3.9 (1.3)	0.05
Average DAS28 year 7-9	166	3.5 (1.2)	28	4.0 (1.3)	0.07
Joint-damage score baseline	221	0 (0-2)	41	0 (0-3)	0.87
Joint-damage score year 3	221	6 (1-15)	41	5 (0-18)	0.70
Joint-damage score year 6	154	13 (2-26)	29	14 (3-26)	0.98
Joint-damage score year 9	109	20 (8-35)	24	20 (3-37)	0.98

DAS28: Disease Activity Score using 28 joint counts. Baseline and follow-up values of disease markers of joint inflammation and bone destruction of 262 RA patients, stratified by *DECTIN-1 *genotype. Values are numbers (percentage), medians (p25-p75) or means (SD), as indicated by the notation.

**Table 3:** Between-group differences for *DECTIN-1 *Y238X genotype in joint-damage progression

Parameter	Estimate	SE	*P *value
Intercept	3.32	0.32	< 0.0001
*DECTIN-1 *genotype	0.05	0.79	**0.95**
			
Intercept	1.07	0.58	0.065
*DECTIN-1 *genotype	-0.40	0.71	**0.57**
Joint damage at baseline	2.65	0.53	< 0.0001
Rheumatoid factor +	1.28	0.63	0.043
Average DAS28^a^	1.26	0.24	< 0.0001

DAS28: Disease Activity Score using 28 joint counts. Results of the linear regression model with 262 RA patients. The upper model tests the difference in annual joint-damage progression rate between wild-type patients and patients heterozygous for the *DECTIN-1 *Y238X polymorphism, indicated by the estimate of *DECTIN-1 *genotype (*P *= 0.95). The lower model tests the same (*P *= 0.57), with addition of baseline joint damage, rheumatoid factor positivity, and the time-averaged DAS28 as confounders. ^a^Time-averaged DAS28 was centered.

## Discussion

Rheumatoid arthritis (RA) is a systemic, chronic inflammatory disorder with autoimmune characteristics that affects 0.5% to 1.0% of the Western population. RA causes progressive cartilage damage and often concomitant bone destruction, which tremendously impairs joint movement. It is generally accepted that a complex interplay of genetic and environmental factors contributes to the etiology of RA.

Dectin-1, a member of the C-type lectin receptor family and the main β-glucan receptor, was recently demonstrated to be involved in promoting pro-inflammatory responses. Dectin-1 synergizes with TLR signalling pathways [11,12] and contributes to induction of T-cell responses, including Th17 [14,29]. Several animal models of experimentally induced arthritis have been shown to be induced or exacerbated by administering fungal-derived particles such as zymosan and glucans that can be recognized by and signal through dectin-1. Moreover, a more specific role for dectin-1 in RA pathogenesis has been investigated in arthritis-prone SKG mice, in which β-glucan induced arthritis could be prevented by competitively blocking the dectin-1 receptor [20].

Very recently, the functional consequences of the Y238X early stop codon polymorphism in *DECTIN-1 *have been studied in detail. This polymorphism was demonstrated to result in a complete loss of function of the protein to bind β-glucan, and, as a consequence, cells homozygous for this polymorphism are unable to induce intracellular signalling and subsequent cytokine production on exposure to β-glucans [21,22].

In this study, dectin-1 and the *DECTIN-1 *polymorphism Y238X (c.714T>G, rs16910526) were examined concerning their role in RA pathogenesis. Dectin-1 mRNA expression was measured with an oligonucleotide expression array and confirmed with quantitative PCR in synovial tissue biopsies from RA patients and compared with OA and nonrheumatic synovial tissue. Dectin-1 mRNA expression was fourfold higher in RA synovial tissue, compared with synovial tissues obtained from OA, in which immune mechanisms are minimally involved, and from nonrheumatic patients. Dectin-1 protein expression in RA synovial tissue was shown to be moderate to high, mostly located on infiltrating macrophage-like cells residing in the synovial sublining and around blood vessels. This indicates that dectin-1 is present in high amounts in RA synovial tissue and therefore can contribute to the inflammatory response exerted by macrophages in this setting.

Subsequently, because dectin-1 appeared to be highly expressed on infiltrating macrophages, the consequences of the *DECTIN-1 *Y238X polymorphism for dectin-1 mediated cytokine production capacity of macrophages were studied. Macrophages from individuals bearing the *DECTIN-1 *polymorphism exhibited an impaired capacity to produce cytokines induced by dectin-1 signalling. This was demonstrated for TNF-α and IL-1β, both crucial cytokines in RA pathogenesis [30,31].

Considering the important consequences for the function of the protein, we analyzed whether the presence of the *DECTIN-1 *Y238X polymorphism is correlated with the susceptibility to and clinical severity of RA in a Dutch cohort of 262 RA patients. An overall allele frequency of 7.8% was obtained and was not significantly different compared with that in a healthy control group (n = 284) with an allele frequency of 7.6% (*P *= 0.87; Table 1). All individuals tested were heterozygous for the polymorphism. The correlation of clinical parameters, that is, inflammation markers and degree of bone destruction, also revealed no statistically significant differences (Table 2). Finding no difference in bone destruction in RA patients homozygous and heterozygous for the *DECTIN-1 *Y238X polymorphism could also be a problem of statistical power. However, the difference we found was nearly zero. With the group sizes we obtained, adopting a two-sided alpha of 0.05, a "power" of 0.80, and an SD of 3 in the usual power calculation formula, we would have been able to detect a difference in annual joint damage progression in a Ratingen score of 1.5, which we regard as reasonably small.

## Conclusions

These data imply that, despite the lower cytokine responses exhibited by individuals heterozygous for the *DECTIN-1 *Y238X polymorphism on stimulation with dectin-1, partial dectin-1 deficiency has a major influence neither on disease susceptibility nor on the degree of inflammation and bone destruction in RA patients. Whether homozygosity for the *DECTIN-1 *Y238X polymorphism may result in a different susceptibility to RA remains to be investigated in studies large enough to identify the rare homozygous individuals.

## Abbreviations

ELISA: enzyme-linked immunosorbent assay; OA: osteoarthritis; PBMCs: peripheral blood mononuclear cells; RA: rheumatoid arthritis; TLR: Toll-like receptor.

## Competing interests

The authors declare that they have no competing interests.

## Authors' contributions

TSP, NT, and RS performed the experiments; JF performed the clinical statistical analysis; TSP, JF, PLvR, WBvdB, MGN, and LABJ designed the study and wrote the manuscript. All authors read and approved the final manuscript.
